# Remarks on the Extensive Use of Purgatives, &c.

**Published:** 1812-12

**Authors:** 

**Affiliations:** Physician to the Fleet, and Inspector of Hospitals, Leeward Islands


*For the Medical and Physical Journal.*

### REMARKS *on the extensive* USE *of* PURGATIVES, *&c.*

*By* DR. DICKSON, *Physician to the Fleet, and Inspector of Hospitals, Leeward Islands.*

THE advantages that have been derived from the administration of purgative medicines, induce me to offer a few observations upon the freedom with which they have been employed on this station.

The attention of practitioners has been so much directed to this subject since the valuable publication of Dr. Hamilton, that the intention, at the present day, must be to add to the weight of evidence already adduced, rather than the hope of conveying new information.

My object is merely to recommend a more extensive trial of this class of remedies to those who have not, by experience, ascertained their good effects; and to observe that a

 febrile febrile state is often kept up by the *retention* of indurated fœces, and other offending matters in the intestines, when they are supposed to have been discharged, because the patient has had two or three partial, loose, evacuations.

In the West Indies I have reason to believe that purgatives have contributed largely to the prevention, as well as to the cure, of disease, in periods of much active service.

Indeed, in tropical climates they are so generally applicable, and even essential, in the treatment of disorders, and particularly fevers, as to constitute a very large portion of medical practice; and the result has been so much in their favor, that, for several years past, I have been inclined to believe that purging, far beyond any other remedy, deserves to be considered the "cure of fevers," as it has been termed by Dr. Wade, whose practical remarks appear to me well entitled to more attention; although, in my opinion, he too generally depreciates the value of blood-letting,—a remedy, certainly, often of great power, if judiciously employed; and particularly during the first hours of fever, or before the morbid actions are fully established.

In the rapid forms of yellow fever, where we have recourse to other powerful auxiliaries, and where the patient, if not saved, is lost in a few hours, the precise value of purging, as an individual remedy, cannot easily be appreciated; but, in those of a modified and more protracted type, I have seen many instances, and I have been favored with many valuable hospital communications, where it has been found extremely difficult to clear the bowels of vitiated bile, mucus, &c. and in which the best effects have resulted from purgatives exhibited not merely with the view of evacuating their feculent contents in the first instance, but in doses repeated from time to time, so as to cause a sufficiently general and continued discharge, until the healthy state of the secretions into the canal returned. Thus in cases in which the sensorium has been much affected, the muscular power depressed, the tongue dry and foul, and the belly obstinately costive, by the free and repeated employment of purgatives, assisted by glysters, the patient has felt relieved, and strengthened, the mouth has become moist, and the digestive and assimilatory functions have resumed their office, in proportion as the intestinal action has been restored. If we trust to the report of the patient, or attendant, however, we are very apt to be deceived in this respect: inspection of the discharges is therefore of great use in enabling us to judge of the state of the canal; and consequently of the freedom with which purgatives will be required so as to ensure their acting through its whole extent, and thus preventing venting any accumulation of feculent matter, or morbid secretions, by which the febrile symptoms may be maintained.

It will not, I presume, be inferred, that I consider extensive purging as always either necessary or proper in fevers; but merely that, although the propriety of evacuating the bowels at the commencement, and of keeping them open during the course of the disease, is universally acknowledged, yet I fear this object is not always accomplished, and, if accomplished, is often far from being sufficient; and that the most evident benefit has resulted from the active exhibition of purgatives, when, sparingly used, they have proved ineffectual.

Illustrative of this, I beg leave to repeat, that I have met with striking instances in which it was supposed that the bowels had been sufficiently acted upon by purgatives, because the patient had had some scanty incomplete dejections; yet, upon having resorted to them again, in consequence of the continuance of the febrile symptoms, a large quantity of indurated fœces, and dark-colored offensive matter, has, at last, been discharged, after which the patient rapidly recovered.

I have also met with some cases of yet greater torpor, where the insensibility of the alimentary canal was such as to require the most powerful remedies of this class to be repeated day after day, to produce effect; and the quantity of dark or black offensive matter thus discharged, and the speediness of its re-accumulation, when intestinal evacuants were not persisted in, would hardly be credited by those who have not witnessed similar instances; the relief that followed, and the recovery of the patient, are the best proofs of the utility of the practice.

With regard to the purgatives to be employed, I need hardly remark, where medicines required to be repeated, and there is a tendency to irritability of stomach, that those should be preferred which can be exhibited in the smallest, and least objectionable, form,—such as jalap, or cathartic extract, with calomel; but the latter should never be trusted to alone, when a few hours are of great consequence, from the uncertainty of its operating, where there is much heat and febrile action.

In the late naval hospital at Fort Royal, Martinique, where intermittents were very prevalent, and frequently accompanied with diseased viscera, arsenic was found far superior to, and had almost entirely superseded, the use of bark.

In a case of tænia which recently occurred in the hospital here, two table spoonfuls of the milky juice of the Carica papaya, papaya, as recommended in the East Indies, and noticed in the Edinburgh Medical and Surgical Journal for January 1811, proved completely effectual. The same success has attended the exhibition of the oil of turpentine: a tape-worm, seventeen feet long, was expelled, last month, a few hours after taking two ounces of this medicine, followed by a dose of castor oil.

*Barbadoes,* D. J. H. DICKSON.

*June 5th,* 1812.

